# Surgical treatment for thyrotoxic hypokalemic periodic paralysis: case report

**DOI:** 10.1186/1477-7819-10-21

**Published:** 2012-01-24

**Authors:** Yi-Chu Lin, Che-Wei Wu, Hui-Chun Chen, Hsiu-Ya Chen, I-Cheng Lu, Cheng-Jing Tsai, Wen-Rei Kuo, Feng-Yu Chiang

**Affiliations:** 1 Department of Otolaryngology - Head and Neck Surgery, Kaohsiung Medical University Hospital, Kaohsiung Medical University, Kaohsiung, Taiwan; 2 Department of Anesthesiology, Kaohsiung Medical University Hospital, Kaohsiung Medical University, Kaohsiung, Taiwan; 3 Department of Nursing, Kaohsiung Medical University Hospital, Kaohsiung Medical University, Kaohsiung, Taiwan; 4 Department of Otolaryngology - Head and Neck Surgery, Faculty of Medicine, College of Medicine, Kaohsiung Medical University, Kaohsiung, Taiwan; 5 Institute of Clinical Medicine, Kaohsiung Medical University, Kaohsiung, Taiwan; 6 Department of Otolaryngology - Head and Neck Surgery, Kaohsiung Municipal Hsiao-Kang Hospital, Kaohsiung Medical University, Kaohsiung, Taiwan

**Keywords:** thyrotoxic hypokalemic periodic paralysis, hypokalemic periodic paralysis, thyrotoxic, thyrotoxic periodic paralysis, thyroidectomy

## Abstract

Thyrotoxic hypokalemic periodic paralysis (THPP) is a rare, potentially life-threatening endocrine emergency. It is characterized by recurrent muscle weakness and hypokalemia. Because many THPP patients do not have obvious symptoms and signs of hyperthyroidism, misdiagnosis may occur. The published studies revealed that definitive therapy for THPP is control of hyperthyroidism by medical therapy, radioactive iodine or surgery, but the long-term post-operative follow-up result was not observed. We reported two cases of medically refractory THPP with recurrent paralysis of extremities and hypokalemia, and both were combined with thyroid nodules. Both patients were treated with total thyroidectomy; the pathology revealed that one is Graves' disease with thyroid papillary carcinoma, and the other is adenomatous goiter with papillary hyperplasia. No episode of periodic paralysis was noted and laboratory evaluation revealed normal potassium level during the post-operative follow up. Our experience suggests that total thyroidectomy by experienced surgeon is an appropriate and definite treatment for medically refractory THPP, especially in cases combined with thyroid nodules.

## Background

Thyrotoxic hypokalemic periodic paralysis (THPP) is a rare endocrine disorder and a reversible medical emergency. The incidence of THPP in Asians, the most frequently affected population, is approximately 2%, while THPP is largely unknown in the West, although the number of reported cases in Western countries has increased recently in the literature [1]. Recent papers show a tendency towards male predominance [2,3]. In Asian populations, THPP usually appears between the ages of 20 and 40 years of life [4], coinciding with the main age for hyperthyroidism.

Hypokalemia with associated flaccid paralysis and signs of hyperthyroidism are the hallmarks of THPP. Hypokalemia occurs due to a massive shift of potassium into the cells rather than net loss from the body. Excess thyroid hormone may predispose to paralytic episodes by increasing the susceptibility of epinephrine or insulin, and therefore increasing Na-K-ATPase activity in the beta-adrenergic receptors in skeletal muscles, which leads to potassium shift into the cells [5].

The published studies revealed that the definitive therapy for THPP is control of hyperthyroidism by medical therapy, radioactive iodine or surgery, but the long-term post-operative follow-up result was not observed. We reported two cases of medically refractory THPP with recurrent paralysis of extremities and hypokalemia, and both were combined with thyroid nodules. After definite treatment by total thyroidectomy, none of them experienced recurrent THPP episodes during long term follow up.

## Case presentation

### Case 1

A 33-year-old male without significant past medical history presented to the emergency department with sudden onset weakness of bilateral lower extremities. This was the third episode in the previous two weeks despite medication. Prior to the episode, the patient had suffered from hand tremors, palpitations, sweating, body weight loss, good appetite, and anxiety for a period of time. His sister had been diagnosed with hyperthyroidism but his family members had no history of similar episodes. After physical examination and laboratory study, the patient was diagnosed with hypokalemic periodic paralysis associated with Graves' disease. After initiation of intravenous potassium replacement, the patient's neurologic symptoms completely resolved.

Reviewing the medical record, the patient received medication for several months, including potassium chloride (600 mg/tid) for hypokalemia, methimazole (30 mg/day) to control his underlying hyperthyroidism, and propranolol (80 mg/day) to control blood pressure and tachycardia. Despite these medications, the patient still suffered from three attack episodes.

We arranged thyroid ultrasonography for this patient, and this revealed focally heterogeneous hypoechoic nodules with calcification, and fine needle aspiration biopsy (FNAB) showed atypia cells. Neck computed tomography (CT) survey also revealed multiple punctuate calcifications in heterogeneous thyroid nodules, which represent an increased risk of thyroid malignancy [6]. Due to FNAB and CT findings, the impression was highly suspected thyroid cancer. Total thyroidectomy was performed for this patient under the impression of Graves' disease with suspected thyroid cancer, and the pathology revealed papillary thyroid carcinoma. After operation, no recurrence of periodic paralysis occurred and laboratory evaluation revealed normal potassium level during the ten years of follow-up period.

### Case 2

A 52-year-old male had past medical history of alcoholic liver cirrhosis and type 2 diabetes mellitus. He suffered from sudden onset generalized weakness especially of the bilateral lower extremities, and he had presented to the emergency department four times in the previous five years. The patient had hand tremors, but he denied any other symptoms and signs of hyperthyroidism. None of his family members had a history of similar episodes or other significant illnesses. The patient was diagnosed with hypokalemic periodic paralysis associated with hyperthyroidism, and the patient's neurologic symptoms completely resolved after intravenous potassium replacement. Thyroid ultrasonography revealed diffuse heterogeneous goiter.

Reviewing the medical record, the patient have received medication for four years, including treatment for hypokalemia with potassium chloride (600 mg/tid), and for thyrotoxicosis with propranolol (80 mg/day) and methimazole (30 mg/day). Despite of these medications, the patient still suffered from recurrent attack episodes. Since thyroid enlargement persisted despite the medication, annual follow-up thyroid ultrasonography revealed diffuse heterogeneous goiter with a left neck lymphadenopathy, and FNAB showed thyroid atypia cells with lymphadenopathy, total thyroidectomy was performed about five years after the initial hypokalemic paralysis attack. Pathology revealed adenomatous goiter with papillary hyperplasia, and lymph node with reactive hyperplasia. After operation, no episode of periodic paralysis was noted and laboratory evaluation revealed normal potassium level during the two years of the follow-up period.

## Discussion

THPP does not usually recur once the patient is euthyroid, except in a few studied cases [2]. Adequate control of hyperthyroidism is the mainstay of therapy. Hsieh et al [7] reported that before achieving the euthyroid status, the rate of recurrent attacks was as high as 62.2%, peaking in the first 3 months after THPP diagnosis. The published studies revealed long-term control treatment for THPP is control of hyperthyroidism by medical therapy with antithyroid drugs [7,8] and nonselective beta-blockers [9,10], radioactive iodine [1,2,8-11] or surgery (thyroidectomy [1,7-10] or subtotal thyroidectomy [11]). Since failure to properly diagnose and treat THPP can be fatal, rapid correction of potassium abnormalities is imperative as it can resolve the symptoms quickly and completely, and definitive treatment depends on the underlying etiology.

In our experience of these two cases, we find total thyroidectomy for treatment of medically refractory THPP may have the following advantages: it can offer a definitive control of hyperthyroidism and the coexisting thyroid disorder, such as Graves' disease or benign/malignant goiter, can also be treated. We believe that surgery is better than radioactive iodine treatment because the latter has the risk of radiation exposure. We also suggest total thyroidectomy rather than subtotal thyroidectomy because of the possibility of recurrence of thyroid tumor which may lead to THPP attack if the patient still has hyperthyroidism, or which may render revision surgery more difficult. However, this procedure should be performed by experienced surgeons to minimize the surgical complications such as recurrent laryngeal nerve injury, hematoma with or without airway obstruction and hypoparathyroidism. Therefore, we suggest that total thyroidectomy by experienced surgeons is an appropriate and definite treatment for THPP.

## Conclusions

We reported two cases of medically refractory THPP; neither of whom suffered from recurrent episodes of paralysis during the long-term follow up after total thyroidectomy. Since failure to properly diagnose and treat THPP can be fatal, rapid correction of potassium abnormalities is imperative as it can resolve the symptoms quickly and completely. Thus, we suggest total thyroidectomy as the definitive therapy for THPP, and the coexisting thyroid disorder, such as Graves' disease or benign/malignant goiter, can also be treated. We believe that total thyroidectomy is better than radioactive iodine treatment due to there being no risk of radiation exposure, and that it is superior to subtotal thyroidectomy due to the lesser risk of thyroid tumor recurrence. Although total thyroidectomy may have certain surgical complications, we suggest that total thyroidectomy by experienced thyroid surgeons is an appropriate and definite treatment for THPP.

## Consent

Patients' written consent was obtained for publication of their case records.

## List of abbreviations

THPP: thyrotoxic hypokalemic periodic paralysis; FNAB: fine needle aspiration biopsy.

## Competing interests

The authors declare that they have no competing interests.

## Authors' contributions

YCL, CWW: Data collection, manuscript composition and submission. HCC, HYC, ICL: Took part in the care of the patient, performed literature search, and helped with manuscript preparation. WRK, FYC: Critical revision and supervision, study conception and design.

All authors read and approved the final manuscript.
